# Electrochemical corrosion properties of a novel sodium hyaluronate and zinc oxide nanoparticle-coated stainless steel orthodontic arch wire – an in-vitro study

**DOI:** 10.2340/biid.v13.46738

**Published:** 2026-08-21

**Authors:** Najiya V. Pallikkara, Shweta Nagesh, Pugalmani Sivashanmugam, Shantha Sundari

**Affiliations:** a Department of Orthodontics, Saveetha Dental College and Hospitals, Saveetha Institute Of Medical And Technical Sciences, Saveetha University, Chennai, India; b Saveetha Dental College and Hospitals, Saveetha Institute of Medical and Technical Sciences, Saveetha University, Chennai, India

**Keywords:** Innovation, corrosion, orthodontic wires, stainless steel, nanoparticles

## Abstract

**Background:**

Nanoparticle-based surface modifications of orthodontic arch wires have been extensively investigated for antimicrobial and frictional properties. However, their effect on corrosion behaviour remains largely unexplored.

**Objectives:**

To evaluate the electrochemical corrosion resistance properties (corrosion potential [Ecorr], polarisation resistance [Rp], corrosion current density [icorr], corrosion rate [CR] and the impedance-derived charge-transfer resistance [Rct]) of zinc oxide and sodium hyaluronate (ZnO-SH)-coated and -uncoated stainless steel (SS) orthodontic arch wire in three simulated pH environments.

**Materials and methods:**

Thirty commercially available 19 × 25” SS arch wires were divided into coated (*n* = 15) and uncoated (*n* = 15) groups. The samples were immersed in neutral, acidic and alkaline pH at 37°C for 2 h. Electrochemical testing included potentiodynamic polarisation and electrochemical impedance spectroscopy (EIS). Statistical comparison was done using Welch’s t-test and two-way Analysis of variance (ANOVA) with Bonferroni-corrected post-hoc comparisons.

**Results:**

The effect of the ZnO–SH coating was strongly environment dependent, with a highly significant coating-to-environment interaction for all rate parameters (*p* < 0.001). In the neutral and alkaline environments, the coated wires showed significantly higher Rp and lower corrosion icorr and CR than the uncoated wires (*p* < 0.001). In the acidic medium, the opposite was observed: the uncoated wires had significantly higher Rp (822 vs. 91 kΩ·cm²) and significantly lower icorr (0.019 vs. 0.273 µA/cm²) than the coated wires (*p* < 0.001). Post-corrosion scanning electron microscopy confirmed an intact coating in the neutral and alkaline environments and extensive coating breakdown, pitting and cavitation in the acidic medium.

**Conclusion:**

The ZnO–SH coating improved the corrosion resistance of SS orthodontic arch wires in the neutral and alkaline but not in the acidic environment.

## Introduction

Nanotechnology has emerged as a transformative force in modern dentistry, altering the way materials are synthesised and used across disciplines [1]. Nanotechnology utilises nanoparticles that are particles in the nanoscale range about 1–100 nm. At this scale, these materials exhibit unique physicochemical, mechanical and biological properties compared to their bulk counterparts owing to a higher surface area to volume ratio [2, 3]. In dentistry, nanotechnology has witnessed rapid development over the past two decades, with nanoparticles incorporated into various dental materials to enhance their properties [4]. In orthodontics, nanoparticles are being utilised primarily for two purposes: antimicrobial and for enhancement of material properties [5]. Among the orthodontic materials, arch wires have attracted particular attention as substrates for nanoparticle-based surface modifications, specifically to reduce microbial biofilm and reduce friction [6, 7].

While the antimicrobial and frictional properties of nanoparticle coatings on orthodontic arch wires have been extensively investigated, a significant research lacunae exist regarding the effect of these coatings on the corrosion behaviour of the arch wires. Corrosion of orthodontic appliances in the oral cavity is a well-documented clinical issue, as the dynamic intra-oral environment characterised by fluctuating pH, enzymatic activity and microbial colonisation can compromise the orthodontic materials used [8, 9]. Karandish et al. [10] evaluated the corrosion resistance of zinc oxide (ZnO)-coated stainless steel (SS) orthodontic arch wires using physical vapour deposition (PVD) and found that the coating improved the electrochemical corrosion properties of the SS wires. Pradeep et al. [11] demonstrated that the surface topography and elemental composition of SS orthodontic brackets were significantly affected by pH variations, with surface degradation and ion release under acidic and alkaline conditions. These findings underscore the relevance of corrosion assessment for any novel surface modification intended for intra-oral use. Hence, it is imperative to assess the electrochemical corrosion characteristics of a novel ZnO and sodium hyaluronate (SH)-coated SS orthodontic arch wire. While ZnO nanoparticle coatings have shown to reduce the number of active anodic sites on the SS arch wire surface, thereby shifting the corrosion potential and decreasing the corrosion density, the integrity of the passive chromium oxide film that inherently protects the SS wires from corrosion remains susceptible to breakdown in response to a harsh oral environment [9, 10]. On the other hand, SH is a naturally occurring glycosaminoglycan, which shows biocompatibility, anti-inflammatory activity and hydration-retentive properties [12]. The synergistic combination of these two materials has not been evaluated on the electrochemical behaviour under simulated oral conditions.

Hence, the aim of the present study was to evaluate the corrosion resistance of novel ZnO-SH-coated SS orthodontic arch wires. The primary objective of the study was to assess the electrochemical corrosion behaviour of ZnO-SH-coated and -uncoated SS wire using open circuit potential (OCP), potentiodynamic polarisation and electrochemical impedance spectroscopy (EIS) across different simulated environments.

## Materials and methods

The study was conducted in vitro. Commercially available 19 × 25” SS orthodontic arch wires (Ormco, USA) were used for the coating process. A total of 30 wire samples were used in the study. They were divided into two groups: Coated (*n* = 15) and uncoated groups (*n* = 15). Convenience sampling was done as it was a preliminary evaluation.

### Sample preparation

ZnO-SH coating was deposited onto the surface of the SS arch wire using PVD system with magnetron sputtering to ensure a uniform and adherent film formation. Prior to coating, the wires were ultrasonically cleaned in ethanol and deionised water to remove surface impurities and then air dried. High-purity ZnO target (99.99%) was sputtered under a base pressure below 1 × 10^−2^ Torr and an argon working pressure of 3 mTorr, with a sputtering power of 120 W. During deposition, SH was co-sputtered simultaneously with ZnO to form a homogenous composite layer. A uniform coating with an approximate thickness of 0.05 µm -0.1 µm was formed. Surface characterisation was done using scanning electron microscopy (SEM).

### Immersion protocol

To simulate intra-oral exposure, both coated and uncoated wires were immersed in three different pH with five samples per medium: neutral medium, prepared using phosphate-buffered saline (PBS; pH 7.0), acidic medium obtained by adding 1N hydrochloric acid adjusting to a pH of 5.5 to artificial saliva and alkaline medium obtained by adding 0.01 M sodium hydroxide to a pH of 9.2 to artificial saliva. The artificial saliva was prepared according to Fusayama’s formulation [13]. All specimens were immersed individually in sealed glass vials containing 5 mL of each medium and incubated at 37°C for 2 h. The 2-h immersion was selected to allow the surface to equilibrate with each medium and the open-circuit potential to stabilise, so that the measurements characterise the early, stabilised corrosion behaviour of the interface. Following immersion, each specimen was rinsed with deionised water and dried before electrochemical evaluation.

### Electrochemical testing

In this study, electrochemical corrosion properties refer to the corrosion potential (Ecorr), polarisation resistance (Rp), corrosion current density (icorr), corrosion rate (CR) and the impedance-derived charge-transfer resistance (Rct). The corrosion behaviour of the coated and uncoated wires was evaluated using potentiodynamic polarisation and EIS. All electrochemical experiments were conducted using an IVIUM A32700 potentiostat system (Ivium Technologies, Netherlands) in a conventional three-electrode configuration, consisting of a SS wire as the working electrode, an Ag/AgCl reference electrode and a platinum mesh counter electrode. To maintain electrical isolation, the distal end of each wire was embedded in self-cure epoxy resin, leaving an exposed surface length of 19 mm in an arch-shaped configuration. Electrical connections were secured using conductive clips to ensure stable contact between the electrode and potentiostat leads.

### Open circuit potential

Prior to polarisation, the OCP of each specimen was recorded for 15 min to ensure potential stabilisation. A stable OCP indicated equilibrium between anodic and cathodic reactions on the wire surface.

### Potentiodynamic polarisation

Potentiodynamic polarisation tests were performed by scanning the potential from −250 mV to +250 mV relative to the stabilised OCP at a scan rate of 1 mV/s, using potential steps of 0.5 mV. The obtained current–potential data were plotted to generate Tafel curves. The corrosion potential (Ecorr) and corrosion current density (icorr) were derived from Tafel extrapolation. The CR (in mm/year) was calculated according to the following relationship:

CR (mm/yr) = 3.27 × 10^−3^ × icorr × E.W. / d

where E.W. is the equivalent weight of the corroding element (g), and d is the density of the alloy (7.9 g/cm³).

### Electrochemical impedance spectroscopy

**Figure 1:**
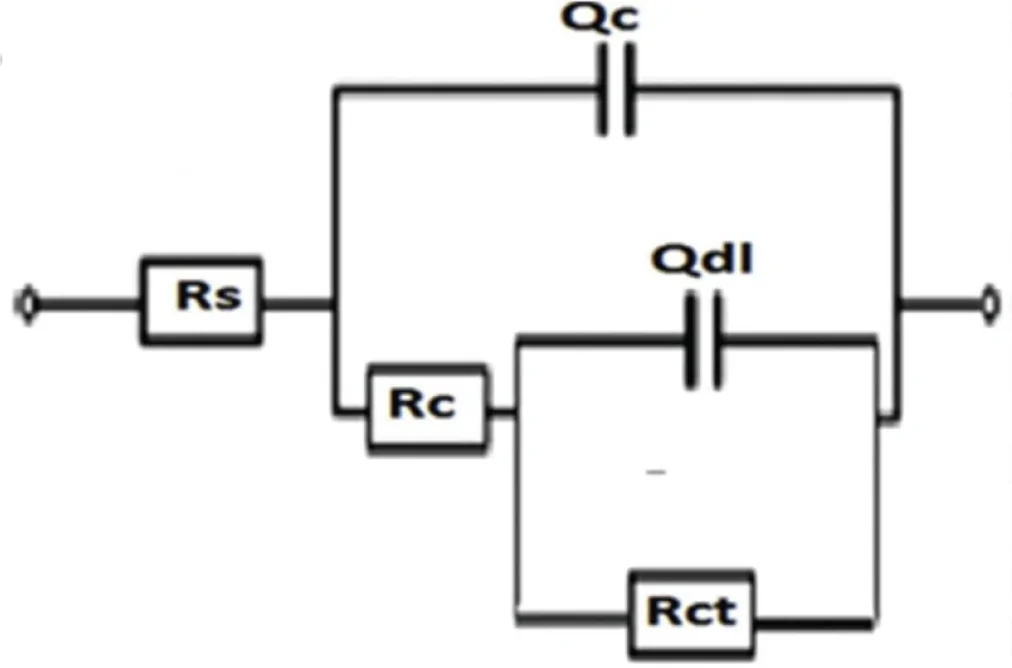
Equivalent electrical circuit model used for fitting the electrochemical impedance spectroscopy (EIS) data. Rs-solution resistance; Qc and Rc-constant phase element and resistance associated with the coating layer respectively; Qdl and Rct – double-layer constant phase element and charge transfer resistance at the metal–electrolyte interface, respectively.

EIS measurements were carried out at the stabilised OCP with a sinusoidal perturbation of ±5 mV amplitude over a frequency range from 100 kHz to 100 mHz. The equivalent circuit model used for fitting the EIS data is presented in Figure 1. The model consists of solution resistance (Rs) in series with two parallel combinations: the coating capacitance (Qc) and coating resistance (Rc) and the double-layer capacitance (Qdl) and charge transfer resistance (Rct). The real (Z′) and imaginary (Z″) components of the impedance were recorded and analysed using ZView and IviumSoft software. A higher Rct value corresponds to improved corrosion resistance due to reduced electrochemical reactivity. All tests were repeated in duplicate for each medium to ensure data reproducibility.

### Surface characterisation

Following electrochemical testing, representative coated and uncoated specimens from each medium were rinsed with deionised water, air dried and examined by SEM to assess coating morphology and post-corrosion surface integrity.

### Statistical analysis

All statistical analyses were conducted using Python (SciPy v1.15). Data are expressed as mean ± standard deviation. Normality was verified using the Shapiro-Wilk test, and homogeneity of variance was assessed using Levene’s test prior to parametric analyses. Comparisons between coated and uncoated groups were made using Welch’s t-test. Because multiple parameters were compared across the three environments, the resulting *p*-values were corrected for multiplicity using the Benjamini–Hochberg false-discovery-rate procedure, and effect sizes (Cohen’s d) were reported alongside each comparison. To assess the combined effects of coating and environment, a two-way factorial ANOVA was performed with coating and medium as fixed factors, followed by Bonferroni-corrected post-hoc comparisons. A *p* < 0.05 was considered statistically significant.

## Results

The OCP measurements stabilised within the first 15 min of immersion for both coated and uncoated groups. Further testing was done following the stabilisation.

### Potentiodynamic polarisation

**Figure 2:**
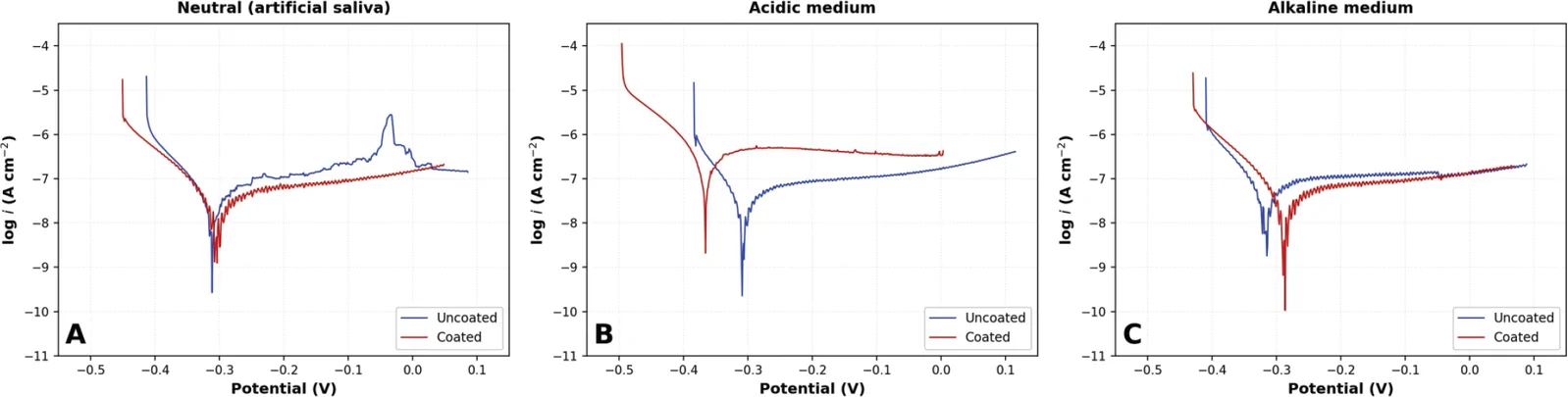
Potentiodynamic polarization curves (Tafel Plots): A: Neutral; B: Acidic; C: Alkaline environments

The potentiodynamic polarisation curves (Tafel plots) for the coated and uncoated groups in the neutral, acidic and alkaline environments are shown in Figure 2, and the extracted parameters are summarised in Table 1. In the neutral medium, the coated group showed significantly higher Rp and significantly lower icorr and CR than the uncoated group (*p* < 0.001), indicating an effective protective coating and the corrosion potential did not differ significantly (*p* = 0.972). In the alkaline medium, the same protective pattern was observed, with the coated group again showing significantly higher Rp and lower icorr and CR (*p* < 0.001). In the acidic medium, however, the opposite was observed, with the uncoated group showing significantly higher Rp and significantly lower icorr and CR than the coated group (*p* < 0.001). The corrosion potential was not significantly different in the acidic medium (*p* = 0.164). Thus, the coating was protective in the neutral and alkaline environments but not in the acidic medium.

**Table 1:** Mean electrochemical parameters (± SD) and statistical comparison (Welch’s t-test) of ZnO–SH coated and uncoated SS orthodontic arch wires across three simulated oral environments.

Environment	Parameter	Uncoated (mean ± SD)	Coated (mean ± SD)	*p*
Neutral	Ecorr (V)	−0.310 ± 0.048	−0.311 ± 0.009	0.972
	βa (mV/dec)	326 ± 16	432 ± 7	< 0.001*
	βc (mV/dec)	−49.3 ± 3.4	−74.0 ± 3.1	< 0.001*
	Rp (kΩ·cm²)	774 ± 5	1368 ± 51	< 0.001*
	icorr (µA/cm²)	0.0480 ± 0.0039	0.0191 ± 0.0009	< 0.001*
	CR (mm/yr)	0.00050 ± 0.00004	0.00020 ± 0.00001	< 0.001*
Acidic	Ecorr (V)	−0.328 ± 0.047	−0.364 ± 0.005	0.164
	βa (mV/dec)	202 ± 6	276 ± 11	< 0.001*
	βc (mV/dec)	−43.1 ± 1.1	−71.8 ± 4.1	< 0.001*
	Rp (kΩ·cm²)	822 ± 11	91 ± 6	< 0.001*
	icorr (µA/cm²)	0.0187 ± 0.0001	0.2734 ± 0.0215	< 0.001*
	CR (mm/yr)	0.00019 ± 0.00000	0.00284 ± 0.00022	< 0.001*
Alkaline	Ecorr (V)	−0.278 ± 0.047	−0.284 ± 0.003	0.780
	βa (mV/dec)	308 ± 6	392 ± 4	< 0.001*
	βc (mV/dec)	−52.8 ± 1.8	−68.1 ± 1.1	< 0.001*
	Rp (kΩ·cm²)	768 ± 5	1121 ± 22	< 0.001*
	icorr (µA/cm²)	0.0460 ± 0.0020	0.0228 ± 0.0009	< 0.001*
	CR (mm/yr)	0.00048 ± 0.00002	0.00024 ± 0.00001	< 0.001*

* *p* < 0.05 – Statistically significant.

Ecorr: corrosion potential; βa: anodic Tafel slope; βc: cathodic Tafel slope; Rp: polarisation resistance; icorr: corrosion current density; CR: corrosion rate; SD: standard deviation; V: volt; mV/dec: millivolts per decade; kΩ·cm²: kiloohm-centimetre squared; µA/cm²: microamperes per centimetre squared; mm/yr: millimetres per year; SS: stainless steel; ZnO–SH: zinc oxide–sodium hyaluronate.

A two-way ANOVA was conducted to evaluate the main effects of coating and medium and their interaction on each electrochemical parameter (Table 2). The medium was a dominant factor for most parameters, and the coating × medium interaction was highly significant for Rp, icorr and CR (*p* < 0.001, η² ≈ 0.52–0.54). This highly significant interaction is the statistical expression of the environment-dependent behaviour: the coating increased Rp and reduced icorr and CR in the neutral and alkaline environments but had the opposite effect in the acidic medium. The main effect of coating was also significant for Rp, icorr and CR (all *p* < 0.001), whereas corrosion potential (Ecorr) showed only a significant effect of medium. Both Tafel slopes (βa, βc) fell within the physically expected range for SS (βc ≈ 43–74 mV/dec; βa ≈ 200–430 mV/dec), the higher slopes of the coated specimens being consistent with the additional charge-transfer barrier imposed by the coating.

**Table 2:** Two-way ANOVA results for Coating × Environment (dferror = 24).

Parameter	Source	*F*	*p*	η²
E_corr	Coating	1.36	0.256	0.030
	Environ.	9.37	< 0.001*	0.410
	C × E	0.78	0.467	0.034
βa	Coating	651.54	< 0.001*	0.341
	Environ.	609.47	< 0.001*	0.638
	C × E	7.46	0.003*	0.008
βc	Coating	539.56	< 0.001*	0.886
	Environ.	6.39	0.006*	0.021
	C × E	16.34	< 0.001*	0.054
Rp	Coating	71.17	< 0.001*	0.008
	Environ.	1928.95	< 0.001*	0.452
	C × E	2286.52	< 0.001*	0.536
i_corr	Coating	423.41	< 0.001*	0.136
	Environ.	518.65	< 0.001*	0.333
	C × E	813.48	< 0.001*	0.523
CR	Coating	423.41	< 0.001*	0.136
	Environ.	518.65	< 0.001*	0.333
	C × E	813.48	< 0.001*	0.523

* *p* < 0.05 – Statistically significant.

C × E = Coating × Environment interaction; ANOVA: analysis of variance; F: F-statistic; η²: partial eta-squared (effect size); df: degrees of freedom; Environ.: Environment; C × E: Coating × Environment interaction; Ecorr: corrosion potential; βa: anodic Tafel slope; βc: cathodic Tafel slope; Rp: polarisation resistance; icorr: corrosion current density; CR: corrosion rate.

### Surface characterisation (SEM)

**Figure 3:**
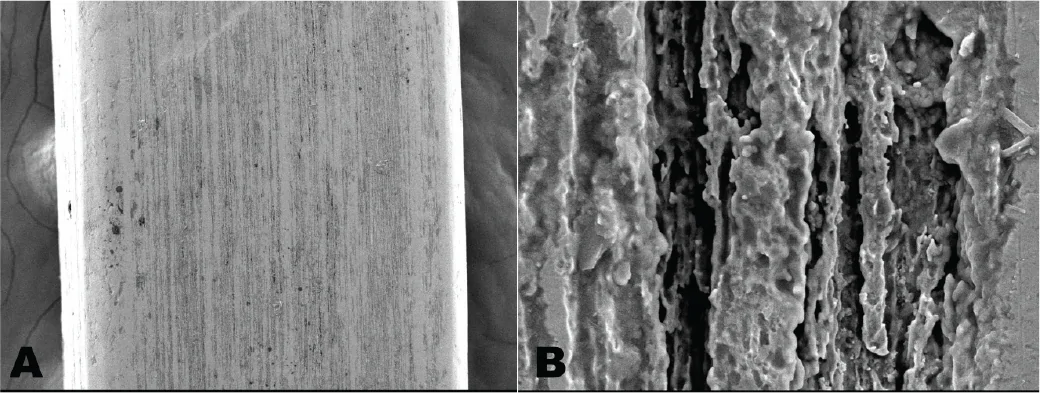
Pre-corrosion scanning electron micrographs of the ZnO–SH coated stainless-steel orthodontic arch wire. (A) Low-magnification overview (50 µm) showing a continuous, uniform coating covering the wire surface; (B) high-magnification view (1 µm) revealing the fine, densely packed nodular morphology of the coating and its homogeneous coverage.

**Figure 4:**
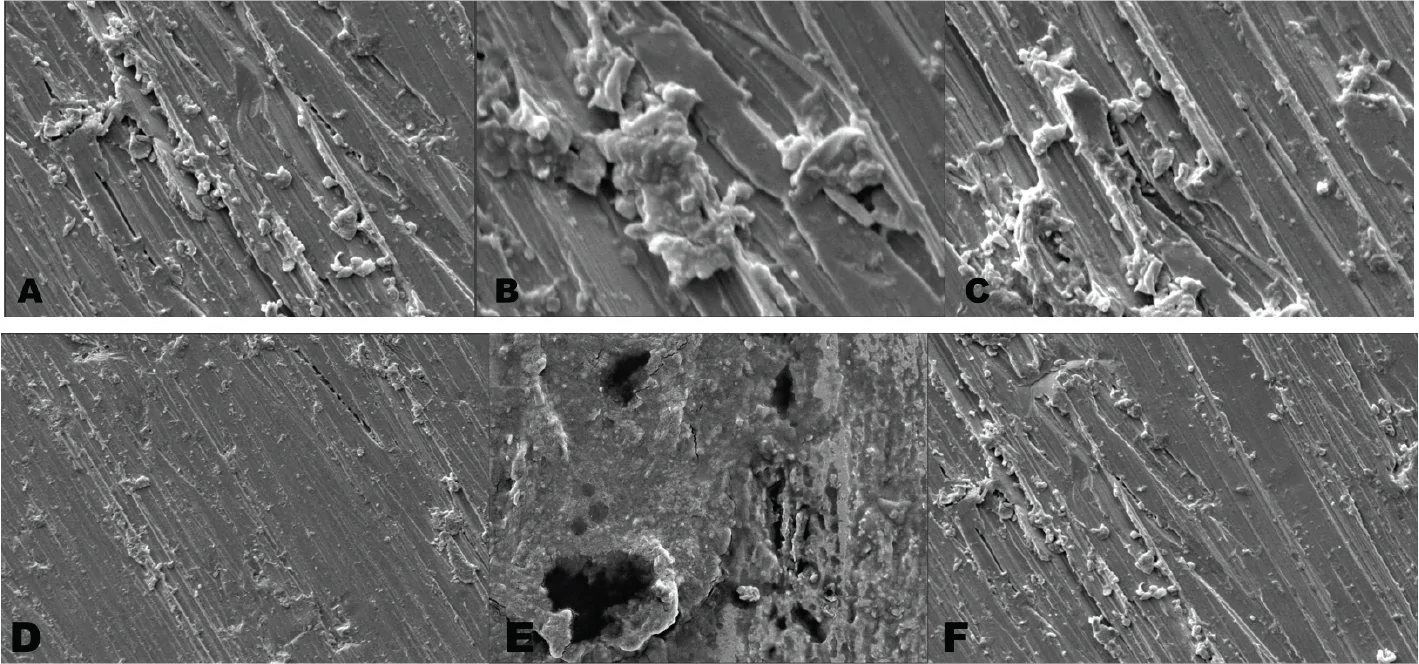
Post-corrosion scanning electron micrographs of uncoated wires in A. Neutral, B. Acidic and C. Alkaline environments, coated wires in D. Neutral, E. Acidic and F. Alkaline environments.

Pre-corrosion SEM of the coated wires showed that the ZnO–SH coating formed a continuous, uniform layer covering the arch-wire surface, following the underlying machining striations (Figure 3A), with a fine, densely packed sub-micron nodular morphology free of cracks or exposed substrate, indicating a homogeneous, well-adhered nanostructured film (Figure 3B). Post-corrosion SEM (Figure 4) corroborated the electrochemical findings. In the neutral and alkaline environments, the coated surfaces were continuous and largely intact, without pitting, whereas the corresponding uncoated surfaces showed the original machining striations with scattered corrosion products. In the acidic medium, the coated surface showed extensive coating breakdown, with pits, cavities and cracking indicating localised attack of the underlying steel, while the uncoated surface remained comparatively intact. These observations provide direct morphological evidence that the coating degrades and loses its protective function.

### EIS

**Figure 5:**
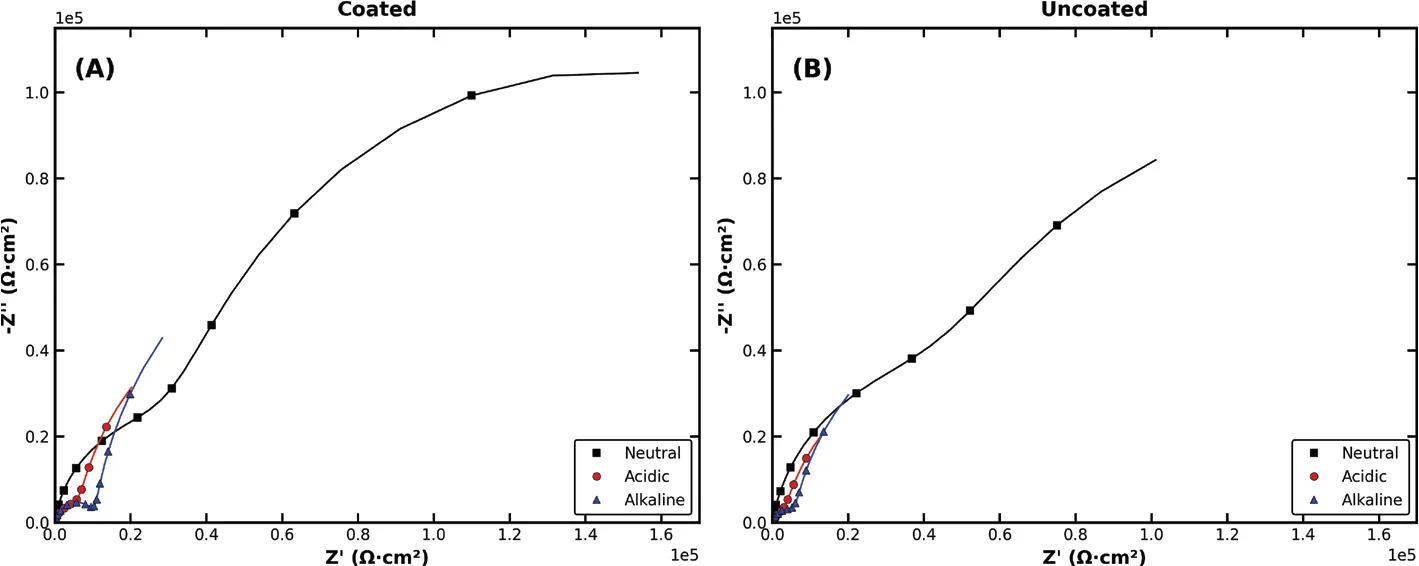
Electrochemical impedance spectroscopy (Nyquist plots)- A: Coated samples; B: Uncoated samples

The EIS was done to further characterise the corrosion behaviour and protective properties of ZnO-SH coating. The impedance response was recorded over a frequency range and is presented as Nyquist plots (Z’ vs. -Z’) for each medium in both coated and uncoated groups (Figure 5). The experimental data were fitted using an equivalent circuit model. The fitted circuit parameters are summarised in Table 3.

**Table 3:** Equivalent circuit parameters obtained from fitting the Nyquist plots for uncoated and ZnO-SH coated orthodontic stainless steel wire in three pH environments.

Parameter	Neutral		Acidic		Alkaline	
	Uncoated	Coated	Uncoated	Coated	Uncoated	Coated
Q_c_ (µS·s^n^) cm^−2^	3.14	1.47	9.14	19.17	6.41	1.17
n_c_	0.88	0.90	0.86	0.82	0.81	0.87
R_c_ (kΩ·cm^2^)	78.40	51.24	9.84	6.14	7.83	11.23
Q_dl_ (µS·s^n^) cm^−2^	11.53	5.17	32.64	41.27	36.17	29.63
n_dl_	0.92	0.93	0.92	0.87	0.91	0.92
R_ct_ (kΩ·cm^2^)	176.14	213.47	117.89	91.47	112.14	172.82

Rs: solution resistance; Qc: constant phase element of the coating layer; Rc: coating resistance; Qdl: double-layer constant phase element; Rct: charge transfer resistance at the metal–electrolyte interface; *n*: CPE exponent (nc for coating, ndl for double layer); µS·s^n^·cm^−2^: micro-siemens-seconds to the power n per centimetre squared; kΩ·cm²: kiloohm-centimetre squared; ZnO-SH: zinc oxide–sodium hyaluronate.

The impedance data were fitted using the two-time-constant equivalent circuit model.

The charge-transfer resistance (Rct) was highest in the neutral medium with 176.14 kΩ·cm² for the uncoated and 213.47 kΩ·cm² for the coated samples (a 21% increase upon coating). In the alkaline medium, Rct increased from 112.14 to 172.82 kΩ·cm² upon coating (a 54% increase). However, in the acidic medium, the Rct was the lowest of the three environments for the coated group (91.47 kΩ·cm² coated and 117.89 kΩ·cm² uncoated). The coating therefore did not provide a reliable impedance benefit in the acidic medium, in agreement with the potentiodynamic polarisation and SEM findings.

## Discussion

The present study evaluated the corrosion resistance of ZnO-SH-coated and uncoated orthodontic SS arch wires in three simulated environments using potentiodynamic polarisation, SEM and EIS. The key finding was that the protective effect of the coating was strongly dependent on the pH of the medium. In the neutral and alkaline environments, the coating significantly increased polarisation resistance and reduced corrosion current density and CR, acting as an effective barrier that restricts the transport of corrosive ions to the metal surface. In the acidic medium, by contrast, the coating did not protect the wire.

The loss of protection in the acidic medium reflects the pH-dependent stability of the zinc oxide phase. ZnO is only sparingly soluble at neutral-to-alkaline pH but dissolves rapidly in acid through proton attack on the oxide surface, releasing Zn^2+^ ions [14]. In the acidic environment, the ZnO component of the coating therefore dissolves, degrading barrier integrity and exposing the underlying steel to localised attack consistent with the pitting and cavitation observed by SEM, whereas at neutral and alkaline pHs, the ZnO remains stable and the coating retains its protective function.

Karandish et al. [10] reported that Zn-coated SS wires exhibited a more positive Ecorr and lower icorr in artificial saliva, with higher Rct for coated versus uncoated wires. The present study demonstrated a comparable trend in the neutral and alkaline environments, with a significant enhancement of Rp upon coating in those environments, suggesting that the addition of SH to the ZnO matrix does not compromise the corrosion-inhibitive properties of the zinc oxide component under neutral-to-alkaline conditions. While both studies confirm that ZnO-based coatings improve the charge transfer resistance in near-neutral saliva, the higher absolute Rct values reported by Karandish et al. may reflect their thicker coating and different artificial saliva formulation, immersion protocol and circuit model, all of which influence the magnitude of fitted impedance parameters.

Another investigation by Nema and Taha [15] compared rhodium-coated NiTi wires and found higher susceptibility to corrosion in acidic mouthwashes compared to uncoated NiTi wires. The present study found an analogous outcome for the ZnO-SH coating. Furthermore, Pulikkottil et al. [16] demonstrated that the polarisation resistance of SS wires in artificial saliva decreased drastically in the presence of 0.5% NaF, highlighting the vulnerability of uncoated SS to fluoride-induced corrosion. As the ZnO–SH coating did not protect the wire at low pH, and fluoride-containing oral products are frequently acidic, its behaviour in a fluoride-containing environment cannot be assumed and remains to be evaluated in future work.

The influence of pH on the corrosion susceptibility of orthodontic wires remains an area requiring further exploration. Castro et al. [9] in their comprehensive review noted that the corrosion of orthodontic wires is strongly related to the acidic environment of the oral cavity and the presence of fluoride ions [17], yet most studies have focused on a single pH condition or compared only two media. Pradeep et al. [11] demonstrated that the surface topography and elemental composition of SS orthodontic brackets were significantly affected by pH variations, with the highest surface degradation scores observed in acidic solutions followed by alkaline and neutral conditions. These findings align with the present study’s observation that environment was the dominant factor controlling electrochemical parameters.

The strengths of the present study include the use of dual electrochemical techniques, which provide complementary information about the corrosion process, the systematic evaluation across three physiologically relevant pH media and post-corrosion surface characterisation by SEM. However, several limitations should be acknowledged. The in vitro design does not fully replicate the complex intra-oral environment, which involves dynamic pH fluctuations, salivary flow, enzymatic activity, microbial biofilm formation and mechanical loading. The 2-h immersion, while sufficient to characterise the early corrosion behaviour, is far shorter than the months-long clinical use of arch wires. The study used a small convenience sample without an a priori power calculation, which required further validation.

Overall, the findings indicate that the ZnO–SH coating is a promising surface modification for orthodontic SS arch wires under neutral-to-alkaline conditions but not under acidic conditions. By reducing the rate of electrochemical corrosion in neutral and alkaline environments, the coating may help mitigate the release of metallic ions such as nickel and chromium from SS wires, which have been associated with hypersensitivity reactions, cytotoxicity and adverse tissue responses [8]. However, its failure at low pH, together with the need for long-term durability data, means that further studies with extended immersion, fluoride-containing and acidic pH, mechanical cycling and in vivo validation are required before clinical recommendation, and formulation strategies to stabilise the ZnO against acid dissolution should be explored.

## Conclusion

The ZnO–SH coated SS arch wires demonstrated better corrosion resistance than uncoated wires in the neutral and alkaline environments. The protective effect of the coating was therefore environment dependent, and the pH was a predominant factor influencing corrosion behaviour.

## Data Availability

The datasets generated and analysed during this study are available from the corresponding author on reasonable request.
